# Large-scale aggregation analysis of eukaryotic proteins reveals an involvement of intrinsically disordered regions in protein folding

**DOI:** 10.1038/s41598-017-18977-5

**Published:** 2018-01-12

**Authors:** Eri Uemura, Tatsuya Niwa, Shintaro Minami, Kazuhiro Takemoto, Satoshi Fukuchi, Kodai Machida, Hiroaki Imataka, Takuya Ueda, Motonori Ota, Hideki Taguchi

**Affiliations:** 1 Cell Biology Center, Institute of Innovative Research, Tokyo Institute of Technology, 4259 Nagatsuta-cho, Midori-ku, Yokohama 226-8503 Japan; 2 Graduate School of Informatics, Nagoya University, Furo-cho, Chikusa-ku, Nagoya 464-8601 Japan; 3 Department of Bioscience and Bioinformatics, Kyushu Institute of Technology, Kawazu 680-4, Iizuka, Fukuoka, 820-8502 Japan; 4 Faculty of Engineering, Maebashi Institute of Technology, 460-1 Kamisadori-machi, Maebashi-shi, 371-0816 Japan; 5 Department of Applied Chemistry, Graduate School of Engineering, University of Hyogo, Himeji, 671-2201 Japan; 6 Graduate School of Frontier Sciences, University of Tokyo, 5-1-5 Kashiwanoha, Kashiwa, Chiba 277-8562 Japan

## Abstract

A subset of the proteome is prone to aggregate formation, which is prevented by chaperones in the cell. To investigate whether the basic principle underlying the aggregation process is common in prokaryotes and eukaryotes, we conducted a large-scale aggregation analysis of ~500 cytosolic budding yeast proteins using a chaperone-free reconstituted translation system, and compared the obtained data with that of ~3,000 *Escherichia coli* proteins reported previously. Although the physicochemical properties affecting the aggregation propensity were generally similar in yeast and *E*. *coli* proteins, the susceptibility of aggregation in yeast proteins were positively correlated with the presence of intrinsically disordered regions (IDRs). Notably, the aggregation propensity was not significantly changed by a removal of IDRs in model IDR-containing proteins, suggesting that the properties of ordered regions in these proteins are the dominant factors for aggregate formation. We also found that the proteins with longer IDRs were disfavored by *E*. *coli* chaperonin GroEL/ES, whereas both bacterial and yeast Hsp70/40 chaperones have a strong aggregation-prevention effect even for proteins possessing IDRs. These results imply that a key determinant to discriminate the eukaryotic proteomes from the prokaryotic proteomes in terms of protein folding would be the attachment of IDRs.

## Introduction

Most proteins must fold into their native structure to exert their function^1^. However, protein folding is a highly complicated process, and many nascent proteins synthesized at the ribosomes are exposed to the risk of forming protein aggregation because of the difficulty of their folding under the physiological conditions^2,3^. To prevent the formation of aggregation, cells have developed molecular chaperones which assist in protein folding and prevent the formation of aggregation in the cell^2–4^. To date, extensive studies have elucidated the mechanism of protein folding and the action of various chaperones^3,5^. However, our knowledge on protein folding is still very limited at a proteome level; previous studies have dealt with only a small handful of well-behaved, “ideal” proteins, meaning that the folding properties of the vast majority of proteins in the cell remain entirely unexplored^6^.

To fill the significant gap in our understanding on folding and aggregation, we previously conducted a comprehensive analysis of protein aggregation by using a chaperone-free reconstituted translation system of *Escherichia coli*, called the PURE system^7,8^. In this analysis, we evaluated the aggregation propensity of more than three thousand *E*. *coli* proteins and revealed the existence of the “aggregation-prone” and “highly soluble” groups and the relationship between the aggregation propensity and several properties such as molecular weight and the relative contents of amino acids. In addition, a comprehensive analysis of aggregation-prevention effects of chaperones revealed that two major bacterial chaperone systems, DnaK/DnaJ/GrpE and chaperonin GroEL/ES, have a global effect to prevent various kinds of aggregation-prone proteins from forming aggregation during protein synthesis^9^. The global aggregation analysis of the *E*. *coli* proteome provided some clues to understand the properties of protein aggregation for a prokaryotic proteome. However, since these findings are limited in the *E*. *coli* proteome, whether these features are universally applicable to proteins from other species, especially to eukaryotic proteins, is still unknown.

It is thought that there are some differences between the prokaryotic and eukaryotic proteome. One of the largest differences is the existence of intracellular organelles such as nucleus, endoplasmic reticulum and mitochondrion. In addition, eukaryotic and prokaryotic cells share a different set of chaperones^3^. In *E*. *coli*, DnaK/DnaJ/GrpE chaperone, a member of Hsp70/40 chaperones, and GroEL/ES, a member of Hsp60/10 chaperones and also known as group I chaperonin, are thought to mainly act in the cytosol^3,10,11^. On the other hand, most eukaryotes have multiple sets of Hsp70/40 chaperones, Hsp90 chaperones, and group II chaperonin CCT in the cytosol^3,12–14^. They are thought to maintain protein homeostasis in the cell by acting cooperatively on the nascent proteins and proteins destabilized by certain environmental changes.

Another major difference between prokaryotic and eukaryotic proteins is assumed to be the existence of intrinsically disordered regions (IDRs). IDRs are frequently found in eukaryotic proteins^15–17^. Some prediction tools estimated that about one-third of eukaryotic proteins have long IDRs^16,18^. Contrary to canonical proteins composed of structural domains^19,20^, IDRs normally do not form specific ordered structures determined by their amino acid sequences, but some assume a tertiary structures only when they are bound to other proteins or ligands^16,21^. Although the fundamental roles of these IDRs for the eukaryotic organisms are not fully elucidated, they are known to constitute a highly complex protein-protein interaction network by their unique binding manner, including the ability to bind multiple binding partners^22,23^.

Here we conducted a comprehensive analysis of aggregation propensity and aggregation-prevention effects of chaperones for more than four hundred *Saccharomyces cerevisiae* cytosolic proteins by using the PURE system. By analyzing the results obtained here and comparing them with the data from thousands of *E*. *coli* proteins reported previously, we attempted to uncover the folding properties of eukaryotic proteins and the differences between prokaryotic and eukaryotic proteins. The results suggest that the physicochemical properties affecting the aggregation propensity are generally common between prokaryotic and eukaryotic proteins, but the proteins that have long IDRs have a strong tendency to form aggregates even though the IDR itself is not the main cause of aggregate formation. In addition, analysis of homologous pairs suggested that the difference in the chaperone set between prokaryote and eukaryote may be associated with the difference in the protein evolution.

## Results

### Comprehensive aggregation analysis for yeast cytosolic proteins

**Figure 1:**
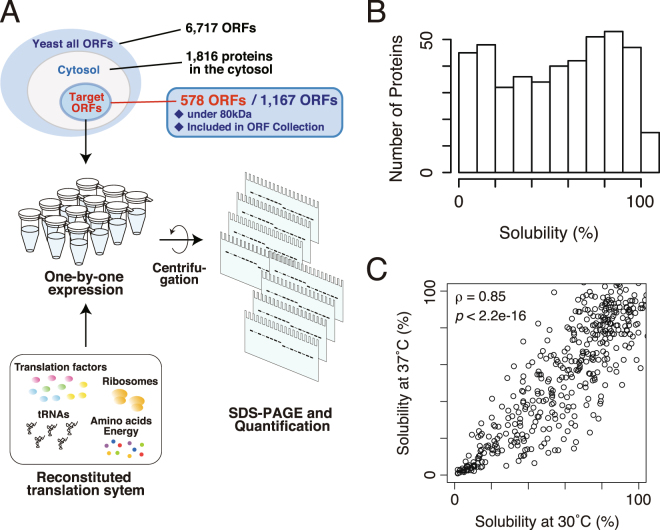
Overview of the experiments and the distribution of the aggregation propensity for yeast cytosolic proteins. (**A**) A schematic illustration of the experiment. Candidate genes were expressed one-by-one by an *E*. *coli* reconstituted translation system (the PURE system). After the synthesis, the aggregation propensities of the synthesized proteins were evaluated by the centrifugation-based method. (**B**) A histogram of the solubility, the index of aggregation propensity, for 447 yeast cytosolic proteins evaluated at 37 °C. (**C**) A scatter plot of the solubility evaluated at 30 °C and 37 °C. The value ρ indicates Spearman’s rank correlation coefficient (*p* < 2.2 × 10^−16^).

We chose *S*. *cerevisiae* proteins as the model of this study due to the wealth of information that is widely available. Unlike prokaryotic cells, eukaryotic cells including *S*. *cerevisiae* have intracellular organelles such as nucleus and mitochondrion. Thus, only the proteins annotated as being located at least in the cytosol were chosen for this analysis. The proteins with other annotations in addition to cytosol (*e*.*g*. nucleus) were included in the target. Among the ~2, 000 proteins annotated to be localized at least in the cytosol, larger proteins (>80 kDa) were omitted because of the difficulty of expression by the PURE system. In addition, proteins that are not included in the purchased ORF collection were also omitted. Among the 1,167 candidates, 578 proteins were finally chosen at random as the target of the analysis. A schematic illustration of the analysis was depicted in Fig. 1A.

We examined the aggregation propensities of all the target proteins by the centrifugation-based assay as previously reported^8,9^. Each protein was expressed one-by-one by the PURE system including [^35^S] methionine for 1 hour at 37 °C. Then, the solubility, which was used as the index of the aggregation propensity, was evaluated by centrifugation at 20,000 × g for 30 min and autoradiography after SDS-PAGE. The reproducibility of the experiment was estimated as the standard deviation of the solubilities were ~10% on average, and the highest standard deviation was 25%, based on data from 33 proteins evaluated by the previous report^8^. Among the 578 tested proteins, the solubilities of 447 proteins were able to be evaluated. The remainder was not quantified due to insufficient translation or trouble during the electrophoresis (translated proteins were stuck in the gel, several protein bands were detected, and so on). Typical examples of these patterns were shown in Supplementary Figure S1A.

A histogram of the quantified solubilities did not show a normal Gaussian distribution (Fig. 1B), indicating the existence of subpopulations with distinct features in the subset of yeast proteins. However, unlike the results of the *E*. *coli* proteins^8^, the solubility distribution was not clearly bimodal (*p* = 0.001, Wilcoxson rank-sum test, Fig. 1B), suggesting that the aggregation-prone properties of *S*. *cerevisiae* cytosolic proteins were somewhat different from those of the *E*. *coli* cytosolic proteome. Overall, the expression yields of the *S*. *cerevisiae* proteins by the PURE system is lower than those of the *E*. *coli* proteins (Supplementary Figure S1B). Correlation between the expression yields by the PURE system and the solubility was weak but positive (Supplementary Figure S1C).

We also evaluated the aggregation propensities of the same set of proteins translated by the PURE system at 30 °C for 3 hours since the optimum growth temperature of *S*. *cerevisiae* is thought to be around 30 °C. Although the distribution of the solubilities under the 30 °C condition biased toward soluble as compared to the solubilities under the 37 °C condition (Supplementary Figure S1D), the distributions evaluated under both conditions showed a strong correlation (Spearman’s rank correlation coefficient ρ = 0.85, *p* < 2.2 × 10^−16^, Fig. 1C). This result suggests that the reaction temperature was not a strong determinant of the aggregation propensity at least under both of the conditions tested.

### Relationship to physicochemical properties and structural information

**Figure 2:**
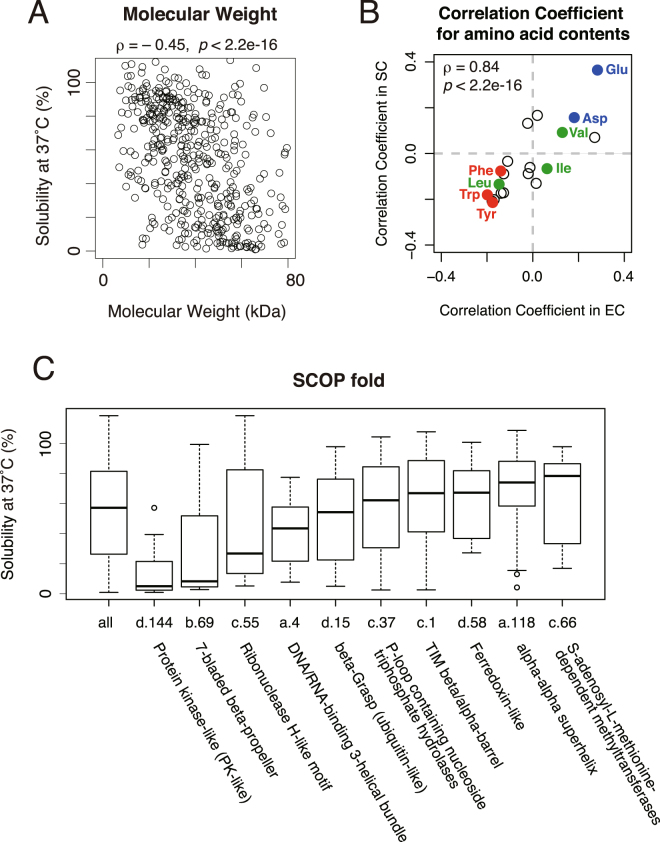
Comparison of the aggregation propensity with physicochemical properties and structural parameter. (**A**) A scatter plot of the solubility evaluated at 37 °C and molecular weight. The value ρ indicates Spearman’s rank correlation coefficient (*p* < 2.2 × 10^−16^). (**B**) A scatter plot of correlation coefficients between the solubility and the ratio of each amino acid residue in the *E*. *coli* (EC) and *S*. *cerevisiae* (SC) proteins. The values in the *E*. *coli* proteome were obtained from the previous report. The value ρ indicates Spearman’s rank correlation coefficient (*p* < 2.2 × 10^−16^). All values were listed in Table S1. (**C**) A boxplot of the solubility evaluated at 37 °C in each SCOP fold group. Only the SCOP folds that contain more than 10 proteins were shown.

We then compared physicochemical properties with the solubility in order to investigate the difference between the aggregation-prone properties of *S*. *cerevisiae* cytosolic proteins and *E*. *coli* cytosolic proteome^8^. As observed in the analysis of the *E*. *coli* proteome, molecular weight and isoelectric point showed a negative correlation with the solubility (Fig. 2A and Supplementary Figure S2A). Also, the correlation coefficients between the solubility and the relative contents of 20 amino acids between the *S*. *cerevisiae* proteins and the *E*. *coli* proteome showed a very similar trend (Fig. 2B and Supplementary Table S1). Furthermore, the hydropathy index (GRAVY score^24^) did not correlate with the solubility, as is the case with the *E*. *coli* proteome (Supplementary Figure S2B)^8^. These results suggest that the physicochemical properties related to the aggregation propensity of *S*. *cerevisiae* cytosolic proteins were largely similar to those of the *E*. *coli* cytosolic proteome.

In the *E*. *coli* proteome, the aggregation propensity showed some correlation to the classification of the Structural Classification of Proteins (SCOP)^8,20^. Then, we annotated SCOP folds with the evaluated proteins and the distributions of the solubility in each fold were compared. The results showed that three folds (d.144; Protein kinase-like, b.69; 7-bladed beta-propeller, and c.55; Ribonuclease H-like motif) were strongly biased to be aggregation-prone. However, most other folds did not show a clear bias toward low or high solubilities. In addition, the proteins categorized in these three folds were significantly enriched in larger molecular weight (Supplementary Figure S2C). Furthermore, some folds categorized to be aggregation-prone in the previous analysis of the *E*. *coli* proteome were relatively biased toward being soluble in this analysis (for example c.1; TIM beta/alpha-barrel and c.37; P-loop containing nucleoside triphosphate hydrolases) (Fig. 2C). These results suggest that the SCOP fold is not a strong determinant for the aggregation propensity for *S*. *cerevisiae* cytosolic proteins.

Comparison of the contents of the secondary structure (coil, helix and strand^25,26^) did not show clear correlations to the solubility (Supplementary Figure S2D). The relationship between the solubility and oligomeric states of proteins showed that the proteins that form heterooligomeric complexes showed biased tendency toward aggregation-prone properties (Supplementary Figure S2E), suggesting that the heterooligomeric proteins tend to form aggregates when their oligomeric partner proteins are absent, although the information about oligomeric states was not sufficient.

### Correlation to amyloidogenic propensity, aggregation prediction, and intrinsically disordered region (IDR)

In yeast cells, some Q/N rich amyloidogenic proteins are known to behave as prions and are thought to be important for the adaptation to various environments^27^. Hence we compared the aggregation propensity and the amyloidogenicity determined by the degree of the enrichment of Q/N residues in 80 amino acid window^27^. The results showed that there was no obvious correlation between them (Supplementary Figure S2F), despite the fact that Q/N rich regions tend to form amyloid aggregates in the cell. We also calculated the predicted aggregation propensities with TANGO algorithm^28^, one of the well-known prediction tools based on the physicochemical principles, and compared the results with the solubility. Again, we could not find any clear correlation between them (Supplementary Figure S2G).

**Figure 3:**
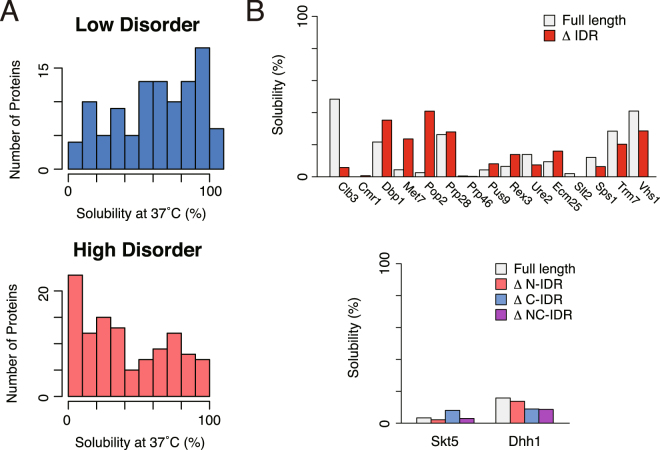
Relationship between the aggregation propensity and intrinsically disordered regions (IDRs). (**A**) Histograms of the solubility evaluated at 37 °C for the proteins in the low and high disorder protein groups. Low and high disorder groups were defined by the length of the longest IDRs that is below the 25^th^ percentile (11 amino acids) and above the 75^th^ percentile (77 amino acids), respectively. The *p* value of Wilcoxon rank-sum test between the two distributions was 1.2 × 10^−6^. (**B**) Solubilities of the IDR-removed proteins. Upper panel shows the solubilities of full-length proteins (white bar) and IDR-removed proteins (red bar) for ten IDR-containing proteins in their N-terminus (Clb3, Cmr1, Dbp1, Met7, Pop2, Prp28, Prp46, Pus9, Rex3, and Ure2) and five IDR-containing proteins in their C-terminus (Ecm25, Slt2, Sps1, Trm7, and Vhs1). Lower panel shows the solubilities of the full-length proteins and IDR-removed proteins in N-terminal (∆N-IDR), C-terminal (∆C-IDR), or both N- and C-terminal (∆NC-IDR) for two model proteins that possess two long IDRs at both N- and C- terminal regions (Skt5 and Dhh1).

Recent researches revealed that many eukaryotic proteins have one or more long unfolded regions, called intrinsically disordered regions (IDRs), which are rarely found in prokaryotic proteins^18^. To investigate the involvement of IDRs in aggregation propensities, we employed DICHOT^29,30^, a prediction tool that classifies regions of a protein sequence into either ordered or disordered groups based on the DISOPRED algorithm^17^. We defined the low disorder group, consisting of the proteins that have only a short IDR (<11 residues, below the lower quartile point), and the high disorder group, consisting of the proteins with long IDRs (>77 residues, above the higher quartile point), and compared their solubility distributions. The results showed that the high disorder group was biased toward the lower solubility fraction, while the low disorder group toward higher solubility fraction (Fig. 3A, *p* = 1.2 × 10^−6^, Wilcoxson rank-sum test). We also investigated the relationship between the solubilities and IDRs by using the ratio of the IDR to the whole amino acid length and obtained a similar tendency (*p* = 0.028, Wilcoxson rank-sum test, Supplementary Figure S3A). These results suggest that the possession of IDRs is associated with the aggregation propensity.

It is known that IDRs have more hydrophilic properties than structured regions, hence IDR itself is expected to be soluble in aqueous solutions^18,22,31^. To investigate whether IDRs are the main cause of protein aggregation, we removed the predicted disordered regions from several proteins that have one or two long IDRs in their N- and/or C-terminal regions, and conducted the aggregation propensity analysis. For this assessment, seventeen aggregation-prone proteins including eight proteins that have homologous counterpart in *E*. *coli* were chosen. The results showed that most of IDR-removed proteins still had high aggregation propensities (Fig. 3B). This result suggests that for most proteins having long IDRs the main cause of aggregate formation is not the property of IDRs, but the folding property of their structured regions. Note that the removed N/C terminal IDRs are not overlapped with the known structural domains. Effects of the removal on the folding are assumed to be slight.

To confirm this notion, we compared the molecular weight of the structural regions with the solubility (Supplementary Figure S3B). The results showed that the molecular size of structural regions and the solubility are negatively correlated irrespective of the existence of IDRs. In addition, two SCOP folds (d.144 and b.69) that showed aggregation-prone properties (Fig. 2C) are strongly enriched in the proteins with longer IDRs (Supplementary Figure S3C). Since these two folds are rarely found in the *E*. *coli* proteome, this result suggests that some eukaryote-specific folds or structures might have aggregation-prone properties, along with the tendency to have longer IDRs.

### Relationship to multiple-localization, essentiality, abundance in cells, and protein functions

It is known that some cytosolic proteins shuttle between nucleus and cytosol when they act. Indeed, about a half of the proteins used in this analysis are annotated to be localized in both the cytosol and the nucleus. Since such multiple-localization behavior of proteins is an eukaryote-specific property and has important roles in cellular function^23^, we investigated the solubility distribution of the proteins that were annotated to be localized in both the cytosol and the nucleus. However, no obvious differences were observed in the solubility distribution (Supplementary Figure S4A). We also investigated the solubility distribution of essential proteins, since essential proteins in the *E*. *coli* proteome showed a significant bias toward higher solubility fraction^8^. However, the solubility distribution of essential proteins in the *S*. *cerevisiae* cytosolic proteins did not show a biased distribution unlike in the *E*. *coli* proteome (Supplementary Figure S4B). Furthermore, a previous report revealed that the solubility of *E*. *coli* proteins positively correlated with their cellular abundance^32^. However, no obvious correlation was observed between the aggregation propensity and cellular abundance in *S*. *cerevisiae* proteins (Supplementary Figure S4C).

Next, to compare the protein function in the cell with the solubility, we classified the proteins by Gene Ontology (GO) classification^33,34^ and compared their solubility distributions. The results showed that some GO categories listed below showed biased solubility distributions toward the aggregation-prone fraction: “transferase activity” and “kinase activity” in the “function” tree, “chromosome” in the “component” tree, and “mitotic cell cycle”, “cellular response to DNA damage stimulus”, “regulation of cell cycle”, “protein phosphorylation”, “organelle fission”, and “mRNA processing” in the “process” tree (Supplementary Figure S4D). However, the proteins in these GO categories tended to be in the high disorder group (Supplementary Figure S4D), suggesting that the direct relationship between the aggregation propensity and protein functions is weak, and this relationship is largely mediated by the existence of long IDRs.

### Analysis of the homologous pairs between *E*. *coli* and *S*. *cerevisiae* proteins

**Figure 4:**
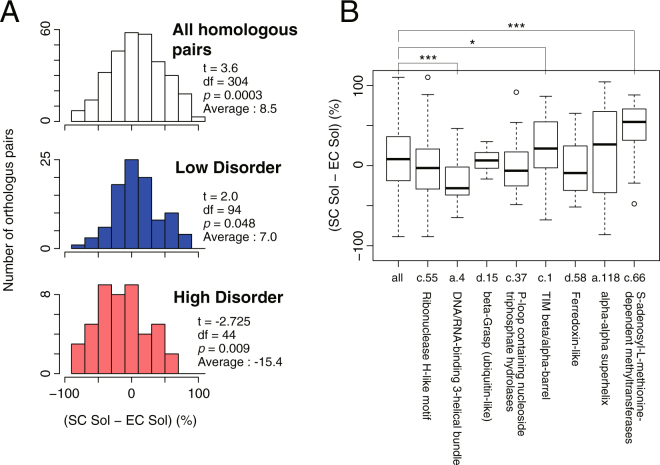
Comparison of the aggregation propensity between the homologous protein pairs in *E*. *coli* and *S*. *cerevisiae*. (**A**) Histograms of the change in the solubility between the homologous protein pairs. The values t, df (degree of freedom), and *p* were calculated by one-sample t-test. (**B**) A boxplot of the change in the solubility between the homologous protein pairs in each SCOP fold group. Two folds (d.144 and b.69) were omitted because the number of protein pairs is too small. *p < 0.05, ***p < 0.01, Wilcoxon rank-sum test.

Among the 447 tested proteins, about one-third of the proteins was found to have homologous proteins in the *E*. *coli* proteome. We then investigated the differences in the solubility between all homologous pairs. The histogram of the differences in the solubility between the homologous pairs indicated that the *S*. *cerevisiae* counterparts tended to have higher solubility than those of *E*. *coli* (Fig. 4A, upper panel). Notably, this tendency was observed only in the low disorder group, whereas an opposite trend was observed in the high disorder group (Fig. 4A, middle and lower panel). These results suggest that *S*. *cerevisiae* proteins that did not have long IDRs tended to be more soluble than *E*. *coli* homologous counterparts, while *S*. *cerevisiae* proteins that have long IDRs showed a stronger tendency to aggregate than the *E*. *coli* counterparts.

We also investigated the relationship between the solubility differences and the SCOP fold (Fig. 4B). The results showed that some folds (c.1; TIM beta/alpha-barrel and c.66; S-adenosyl-L-methionine-dependent methyltransferases) tend to have higher solubility in *S*. *cerevisiae* counterparts, whereas another fold (a.4; DNA/RNA-binding 3-helical bundle) showed an opposite trend (Fig. 4B). These results suggest that the solubility difference between *S*. *cerevisiae* and *E*. *coli* homologous pairs has some relationship with the structural properties such as the SCOP folds. However, these trends might be partially attributed to the possession of long IDRs, since the former two folds (c.1 and c.66) and the latter fold (a.4) were enriched in the low disorder, and the high disorder groups, respectively (Supplementary Figure S3C).

### Large-scale analysis of chaperone effects

In our previous report, most aggregation-prone proteins in *E*. *coli* were found to be rescued by one or the combination of the three major chaperones; Trigger Factor, DnaK/DnaJ/GrpE, and GroEL/ES^9^. *S*. *cerevisiae* cytosol also has many kinds of chaperones, including multiple Hsp70/40 systems and group II chaperonin CCT. Therefore, the aggregation-prone proteins in *S*. *cerevisiae* cytosol were expected to be solubilized by these chaperones.

Before the assessment of the aggregation-prevention effect of chaperones, we investigated the solubility distribution of known chaperone substrates/interactors in *S*. *cerevisiae*. The substrates of CCT^35^ showed a strong tendency to aggregate, although the number of the substrates was small (Supplementary Figure S5A). In contrast, the interactors of Ssa1 and Ydj1^36^, the orthologs of the bacterial DnaK/DnaJ, did not show any biases in the solubility distribution (Supplementary Figure S5B).

**Figure 5:**
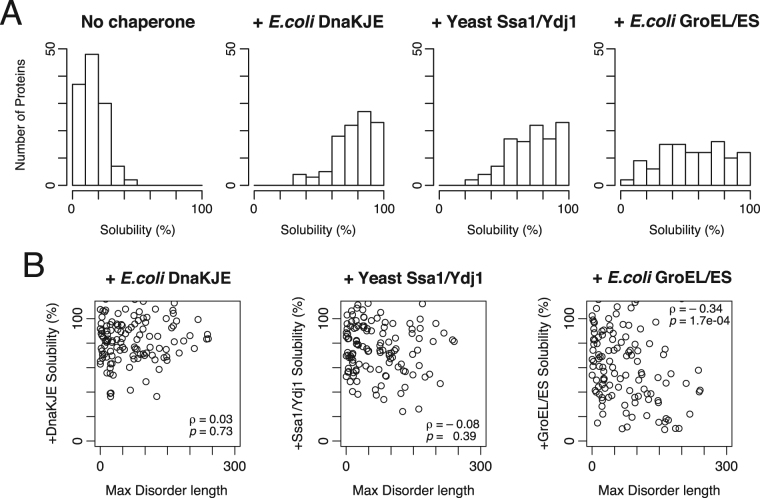
Aggregation-prevention effects of the bacterial and eukaryotic chaperones on the aggregation-prone proteins. (**A**) Histograms of the solubilities in the absence of chaperones or in the presence of each chaperone system for 124 aggregation-prone proteins. The solubilities were evaluated at 37 °C. (**B**) Scatter plots between the longest IDR length and the solubility in the presence of each chaperone system. The value ρ indicates Spearman’s rank correlation coefficient (*p* = 0.73 for the data of *E*. *coli* DnaKJE, *p* = 0.39 for the data of yeast Ssa1/Ydj1, and *p* = 1.7 × 10^−4^ for the data of *E*. *coli* GroEL/ES).

We then investigated the aggregation-prevention effects of eukaryotic Hsp70/40 chaperone, Ssa1/Ydj1 from *S*. *cerevisiae* and two bacterial chaperone systems; bacterial Hsp70/40, DnaK/DnaJ/GrpE and bacterial group I chaperonin GroEL/ES. Among the 447 tested proteins, 124 aggregation-prone proteins (defined as the proteins less than 30% solubility) were selected, and we subsequently evaluated their solubilities in the presence of each of the three chaperones at 37 °C. The distribution of the solubilities showed that both Hsp70/40 chaperones, Ssa1/Ydj1 and DnaK/DnaJ/GrpE, can solubilize a wide spectrum of aggregation-prone proteins (Fig. 5A). In contrast, the effect of GroEL/ES was relatively weak compared to Hsp70/40 chaperones. We found that the solubility in the presence of GroEL/ES negatively correlated with the max IDR length, suggesting that the bacterial GroEL/ES tend to disfavor the proteins with long IDRs (Fig. 5B). On the other hand, both Hsp70/40 chaperones exert a strong aggregation-prevention effects regardless of the presence of IDRs (Fig. 5B). These results suggest that both bacterial and yeast Hsp70/40 chaperones can rescue various kinds of aggregation-prone proteins including eukaryotic-specific long IDR-attached proteins, while the group I chaperonin GroEL/ES tends to prefer bacterial-type proteins containing fewer IDRs. In addition, the aggregation-prevention effects of chaperones did not correlate with molecular weight (Supplementary Figure S5C), suggesting that the influence of molecular size on the chaperone effects is small.

We also evaluated the aggregation-prevention effect of eukaryotic group II chaperonin CCT from human. The results showed that CCT showed a limited aggregation-prevention effect (Supplementary Figure S5D) unlike other chaperones tested here. This result suggests that CCT cannot act solely on nascent polypeptides unlike GroEL/ES^37,38^ and might need other upstream chaperones such as prefoldin, Hsp70/40 systems and ribosome-associated chaperones^3^. Alternatively, CCT may have a relatively strong substrate specificity among the species.

## Discussion

In this analysis, we conducted a large-scale aggregation analysis of eukaryotic cytosolic proteins by using a reconstituted cell-free translation system. Although the translation of eukaryotic ORFs in the PURE system composed of *E*. *coli* translation factors does not fully reconstitute the physiological condition, our aim is to understand the broad-scale trends of protein folding in other organisms by comparing with those of *E*. *coli*. In this context, the PURE system is an ideal tool to conduct the analysis since such a large amount of the chaperone-free translation reaction mixture is currently only available in *E*. *coli*.

Because the PURE system only consists of prokaryotic translational factors^7^, one might think that the aggregation propensity of the evaluated proteins may be affected due to the translation speed differences between eukaryotes and prokaryotes. We cannot completely rule out this possibility, but we reason that the influence of the difference in the translation speed is limited because the solubilities evaluated under the temperature conditions at 30 °C and 37 °C were strongly correlated (Fig. 1C). If the effect of the difference in the translation speed was dominant, the drastic changes would be observed in the solubilities evaluated by the 30 °C and 37 °C experiments. This notion is also supported by the fact that the *E*. *coli* proteins and the *S*. *cerevisiae* cytosolic proteins shared the physicochemical properties connecting to the aggregation propensity (Fig. 2A and B, and Supplementary Figure S2A and B).

The physicochemical properties related to the aggregation propensity were generally common between the *E*. *coli* and the *S*. *cerevisiae* cytosolic proteins (Fig. 2A and B, and Supplementary Figure S2A and B)^8^. These results suggest that the nature of aggregate formation is fundamentally comparable irrespective of the derived species; larger molecular weight proteins tend to form aggregates, and relative contents of negatively charged amino acids and aromatic amino acids are correlated with the aggregation propensity. On the other hand, the relationship of the structural features (the SCOP folds) to the aggregation propensity was not obvious in the *S*. *cerevisiae* cytosolic proteins (Fig. 2C and Supplementary Figure S2C), whereas the structural features had a strong relationship in the previous analysis of *E*. *coli* proteins^8^. This discrepancy suggests that the properties of the fold itself are not the dominant factor for aggregation propensity. It also suggests that the dependency on the structural properties may differ between prokaryotic and eukaryotic cytosolic proteins. For example, two of the three folds that showed an aggregation-prone tendency are eukaryote-specific folds, suggesting that eukaryote-specific structural features affecting the aggregation propensity might exist.

Another key finding is that the proteins containing long intrinsically disordered regions (IDRs) showed a biased solubility distribution toward aggregate formation (Fig. 3A). IDRs primarily have hydrophilic properties^16,22,31^ and hence are unlikely to contribute to the biased aggregation-prone propensity. Therefore, the main reason for this tendency would be due to the aggregation-prone properties of the structural region of the proteins. In fact, the solubilities in some proteins did not change when their IDRs were removed (Fig. 3B). Furthermore, the solubilities of the proteins with long IDRs tended to be lower than those of the homologous counterparts in *E*. *coli* (Fig. 4A). These results suggest that the aggregation propensity of the proteins with long IDRs tends to remain unaltered or even to increase during evolution.

In contrast, the proteins that only have short IDRs showed a biased distribution toward high solubility, and tended to be more soluble than their homologous counterparts in *E*. *coli* (Figs 3A and 4A). These results suggest that the proteins in the low disorder group, which are regarded here as “prokaryote-type” proteins, were evolved to be more soluble compared to the proteins that have longer IDRs. These “prokaryote-type” proteins contain many metabolic enzymes including the proteins having TIM beta/alpha-barrel (c.1) fold, which are known to be favored by GroEL/ES in *E*. *coli*^10^. Hence, the lack of group I chaperonins like GroEL/ES in the cytosol might be associated with the evolutionary processes of the protein folding for these “prokaryote-type” proteins in eukaryotic cytosol, assuming that CCT, a group II chaperonin, does not play a role to substitute the function of group I chaperonins. This notion is supported by the fact that the homologs having c.1 fold in *S*. *cerevisiae* tend to have higher solubility (Fig. 4B).

Analysis of chaperone effects revealed a difference in chaperone preferences; both *E*. *coli* and yeast Hsp70/40 chaperones showed a strong aggregation-prevention effect on a variety of proteins, whereas GroEL/ES showed a limited effect (Fig. 5A), which could be partly attributed to a low preference of GroEL to the proteins with long IDRs (Fig. 5B). In addition, the two folds (c.1 and c.66) in which the *S*. *cerevisiae* homologs showed a higher solubility are frequently found in the GroE class III substrates in *E*. *coli*^10^, while the folds showing an opposite trend (a.4 and c.37) are enriched for the strong interactors with DnaK in *E*. *coli*^11^ (Fig. 4B). Furthermore, the former two folds tend to be in the low disorder group than the latter two folds (Supplementary Figure S3C). From these findings and notions described in the preceding paragraph, we consider the evolution of protein folding in the eukaryotic cytosol as follows: the proteins not having long IDRs, especially for the proteins with severe folding defect like the substrates of GroEL/ES, tend to evolve to be soluble possibly because of the lack of group I chaperonins as described above, while the proteins having long IDRs can still be aggregation-prone because of the assistance for folding by more versatile Hsp70/40 chaperone systems in eukaryotes. Of course, it is difficult to conclude a causal relationship between the protein evolution and the chaperone loss or development. In any case, the evolution of protein folding could be associated with the properties of the chaperone sets in the cellular environment. Our results suggest that such IDR-mediated functions in eukaryote proteomes may be largely maintained by the eukaryotic chaperone sets. In other words, the eukaryotic chaperone sets may have permitted more complicated protein interaction networks provided by various IDRs, which have been developed in the long evolutionary process.

Although our analysis has several limitations and the reality of protein evolution is expected to be much more complicated, our results provide a unique resource to uncover a part of the mystery of protein folding and evolution. Moreover, our “reconstituted proteome” approach, in which each of the properties of hundreds of thousands of proteins are accumulated and analyzed statistically, has a great potential to understand the nature of various proteomes in the protein universe.

## Methods

### Template DNA for cell-free translation

For the expression of 578 *S*. *cerevisiae* cytosolic proteins, we used the ORF collection of *S*. *cerevisiae* commercially available (provided by Open Biosystems, which is a part of GE Healthcare Inc.)^39^. All the ORFs were cloned into the pBG1805 plasmid vector. Each template DNA was amplified by 2-step PCR reaction with common primer sets. The sequences of the primers were as follows; Primer_Fw1: AGACCACAACGGTTTCCCTCTAGAAATAATTTTACAAGTTTGTACAAAGGAGAAGGCTACAAAATG, Primer_Fw2: GAAATTAATACGACTCACTATAGGGAGACCACAACGGTTTCCCTCTAG, Primer_Rv1: GTTATTGCTCAGCGGCAACCACTTTGTACAATTAAGCTGG. The first PCR was conducted with Primer_Fw1 and Primer_Rv1 and the second PCR was conducted with Primer_Fw2 and Primer_Rv1. For the expression by a reconstituted cell-free translation system, Primer_Fw1 contains SD sequence and start codon (underlined), Primer_Fw2 contains the T7 promoter sequence (underlined), and Primer_Rv1 contains UAA stop codon (underlined). The template DNA for the expression of the IDR-truncated proteins were prepared by site-directed mutagenesis with PrimeSTAR Max DNA polymerase (Takara Bio Inc., Japan).

### Preparation of cell-free translation system and chaperones

Preparation of a reconstituted translation system (the PURE system^7^) was described as previously reported^8^. All chaperones except yeast Ssa1 and human CCT were expressed in *E*. *coli* and purified by the following procedures. Hexahistidine-tagged, DnaK, DnaJ, GrpE, and GroES were purified by metal-chelating chromatography and ion exchange chromatography according to the previous report^9^. GroEL was prepared by hydrophobic interaction chromatography and size exclusion chromatography as previously reported^40^. Yeast Ydj1 was purified by anion exchange chromatography and hydroxyapatite chromatography according to the previous report^41^. Hexahistidine-tagged yeast Ssa1 was expressed in *S*. *cerevisiae* and purified according to the previous report^41^. Human CCT was purified from HeLa cells according to the previous report^42^. The concentration of each chaperone during the translation reaction was as follows: DnaK, DnaJ, and GrpE: 5.0, 2.0, and 2.0 µM respectively, yeast Ssa1 and Ydj1: 5.0 and 5.0 µM respectively, GroEL and GroES: 0.5 and 1.0 µM (as tetradecamer and heptamer) respectively, human CCT: 0.5 µM (as hexadecamer).

### Cell-Free Protein Synthesis and Centrifugation- Based Aggregation Assay

Translation reaction and following aggregation evaluation assay were performed as reported previously^8^. In brief, translation reaction was performed at 37 °C for 1 hour or at 30 °C for 3 hours by the PURE system containing [^35^S] methionine. After the reaction, Total and Sup fractions were prepared by centrifugation at 20,000xg for 30 min. The intensities of each band were quantified by autoradiography after SDS-PAGE (FLA7000 fluoroimager and Multi Gauge software, Fujifilm, Japan). The ratio of the intensities of the supernatant (Sup) to uncentrifuged (Total) fractions was defined as the solubility, the index of protein aggregation propensity. For some of the IDR-removed experiments, N-terminal fluorescent label method by using a pre-charged Cy5-Met-tRNA^fMet^ was used instead of [^35^S] methionine labeling^43^.

### Data Analyses

The annotation of subcellular locations was obtained from Yeast GFP Fusion Localization Database (http://yeastgfp.yeastgenome.org/)^44^. The information on molecular weight, isoelectric point, amino acid content, and GRAVY score (the index of hydrophobicity) was obtained from *Saccharomyces* Genome Database (http://www.yeastgenome.org)^45^. The SCOP classification was determined by homology search using PSI-BLAST^46^ against domain sequences in SCOP (Version 2.03)^47^. Position specific scoring matrices were complied based on NCBI NR database (the maximum number of iteration = 4). The results of secondary structure prediction (PSIPRED^25,26^) were provided by GTOP database (http://spock.genes.nig.ac.jp/~genome/gtop.html)^48^. The oligomeric states of proteins were referred to SUBUNIT annotation in UniProt database (http://www.uniprot.org)^49^. The definition of Q/N rich regions was followed by the report by Michelitsch and Weissman^27^, and the calculation was conducted by in-house Perl scripts. The predicted aggregation propensity was calculated by TANGO 2.3^28^, whose binary program was obtained from the website (http://tango.crg.es/). The prediction of intrinsically disordered regions was performed by DICHOT algorithm^29,30^. Essentiality of each gene was obtained from the report by Giaever *et al*.^50^. Protein abundance in the cell was obtained from the report by Huh *et al*.^51^. The enrichment analysis based on Gene Ontology classification^33,34^ was conducted by GO slim mapper provided by *Saccharomyces* Genome Database^45^. The homologous pairs between *E*. *coli* and *S*. *cerevisiae* proteins were defined based on the database of Clusters of Orthologous Groups of proteins (COGs)^52^. ClustalW2 software was used to calculate the amino acid identity score between an homologous pair. The amino acid sequences were downloaded from the Kyoto Encyclopedia of Genes and Genomes (KEGG) database^53^ according to the gene identifiers of the COG database. The CCT substrates and the interactors of Ssa1 and Ydj1 were obtained from the reports by Yam *et al*.^35^ and Gong *et al*.^36^. All the statistical analyses were conducted by the R software (version 3.3.3; http://www.R-project.org).

## Electronic supplementary material

Supplementary information

Supplementary Dataset S1

Supplementary Dataset S2

Supplementary Dataset S3
