# Supplementary material for: Large-scale aggregation analysis of eukaryotic proteins reveals an involvement of intrinsically disordered regions in protein folding
Source: Sci Rep. 2018 Jan 12;8:678. doi: 10.1038/s41598-017-18977-5 (PMC5766493; doi:10.1038/s41598-017-18977-5)
Supplement: Supplementary file 1 — Supplementary information [file 41598_2017_18977_MOESM1_ESM.pdf]

Supplementary Data for:

Large-scale aggregation analysis of eukaryotic cytosolic proteins reveals an involvement of intrinsically disordered regions in protein folding

Eri Uemura[1], Tatsuya Niwa[1], Shintaro Minami[2], Kazuhiro Takemoto[3], Satoshi Fukuchi[4], Kodai Machida[5], Hiroaki Imataka[5], Takuya Ueda[6], Motonori Ota[2], and Hideki Taguchi[1,*]

[1] Cell Biology Center, Institute of Innovative Research, Tokyo Institute of Technology, 4259 Nagatsuta-cho, Midori-ku, Yokohama 226-8503, Japan

[2] Graduate School of Informatics, Nagoya University, Furo-cho, Chikusa-ku, Nagoya 464-8601, Japan

[3] Department of Bioscience and Bioinformatics, Kyushu Institute of Technology, Kawazu 680-4, Iizuka, Fukuoka 820-8502, Japan

[4] Faculty of Engineering, Maebashi Institute of Technology, 460-1 Kamisadori-machi, Maebashi-shi 371-0816, Japan

[5] Department of Applied Chemistry, Graduate School of Engineering, University of Hyogo, Himeji 671-2201, Japan

[6] Graduate School of Frontier Sciences, University of Tokyo, 5-1-5 Kashiwanoha, Kashiwa, Chiba 277-8562, Japan

[*] To whom correspondence should be addressed. Email: taguchi@bio.titech.ac.jp

Supplementary Data included:

- **Supplementary table**
- **Supplementary figures (5)**
- **Supplementary datasets (3)**

Supplementary Table S1: Correlation Coefficients between the solubility and the ratio of each amino acid content.

Amino Acid	With SC solubility (this work)		With EC solubility (Niwa *et al. PNAS* 2009)	
	Spearman's rho	*p*-value	Spearman's rho	*p*-value
Ala	0.17	4.5.E-04	0.02	0.336
Arg	-0.21	5.7.E-06	-0.18	1.E-17
Asn	-0.08	0.080	-0.02	0.342
Asp	0.16	9.E-04	0.18	3.E-18
Cys	-0.17	3.E-04	-0.14	4.E-11
Gln	-0.04	0.412	-0.11	1.E-07
Glu	0.36	3.E-15	0.28	4.E-43
Gly	0.13	0.006	-0.02	0.286
His	-0.20	3.E-05	-0.17	5.E-16
Ile	-0.07	0.131	0.06	0.003
Leu	-0.13	0.004	-0.15	1.E-12
Lys	0.08	0.114	0.27	1.E-39
Met	-0.07	0.162	-0.01	0.562
Phe	-0.08	0.094	-0.14	2.E-11
Pro	-0.09	0.072	-0.13	1.E-09
Ser	-0.17	4.E-04	-0.13	9.E-10
Thr	-0.14	0.004	0.01	0.482
Trp	-0.18	2.E-04	-0.20	1.E-21
Tyr	-0.21	5.E-06	-0.18	3.E-17
Val	0.09	0.065	0.13	6.E-10

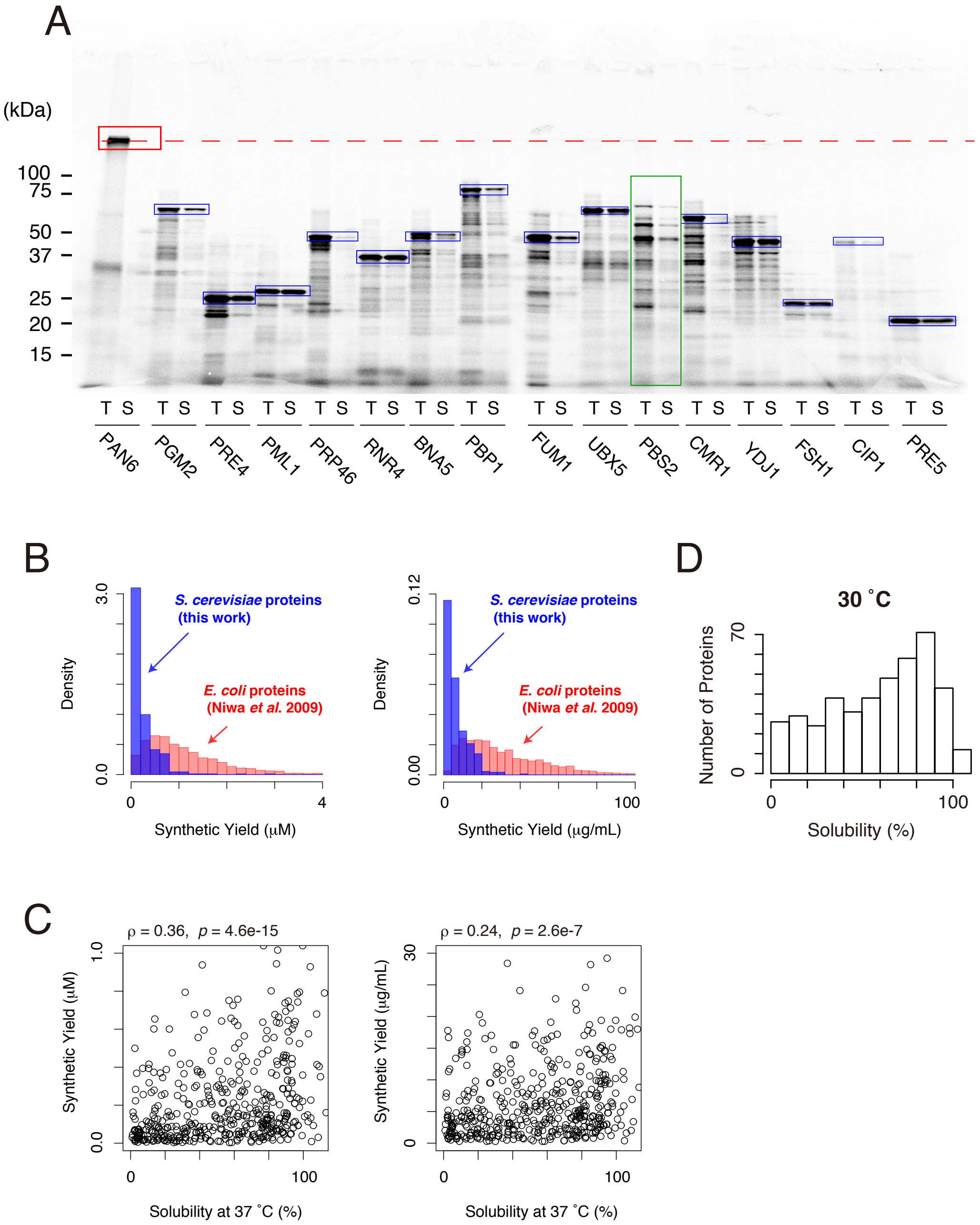

Supplementary Figure S1. An example of the SDS-PAGE pattern, a relationship with the synthetic yields by the PURE system, and a distribution of the aggregation propensity evaluated at 30 °C.
(A) An example of the raw SDS-PAGE pattern. Blue squares indicate the bands used for the calculation of the solubilities. A red dashed line indicates a boundary between the stacking gel and the separating gel and a red square shows a stuck at the boundary. In a green square, a main band could not be determined due to the presence of many extra bands, which were presumably generated by immature translation abortion. In this pattern, PAN6 and PBS2 were excluded from the evaluation of the solubility. (B) Comparison of the distribution of the synthetic yields of *S. cerevisiae* proteins and of *E. coli* proteins translated by the PURE system. Synthetic yields were calculated with the intensity of [^{35}S] methionine. The yields on *E. coli* proteins are from our previous dataset[8]. The yields are expressed as molar concentration (*left*) derived from theoretical molecular weights and as μg/mL (*right*). (C) Scatter plots of the synthetic yield by the PURE system against the aggregation propensity evaluated at 37 °C. The value ρ indicates Spearman's rank correlation coefficient ($p = 4.6 \times 10^{-15}$ for the data of synthetic yield calculated as μM and $p = 2.6 \times 10^{-7}$ for the data calculated as μg/mL). (D)

A histogram of the solubility for 420 yeast cytosolic proteins translated by the PURE system at 30 °C.

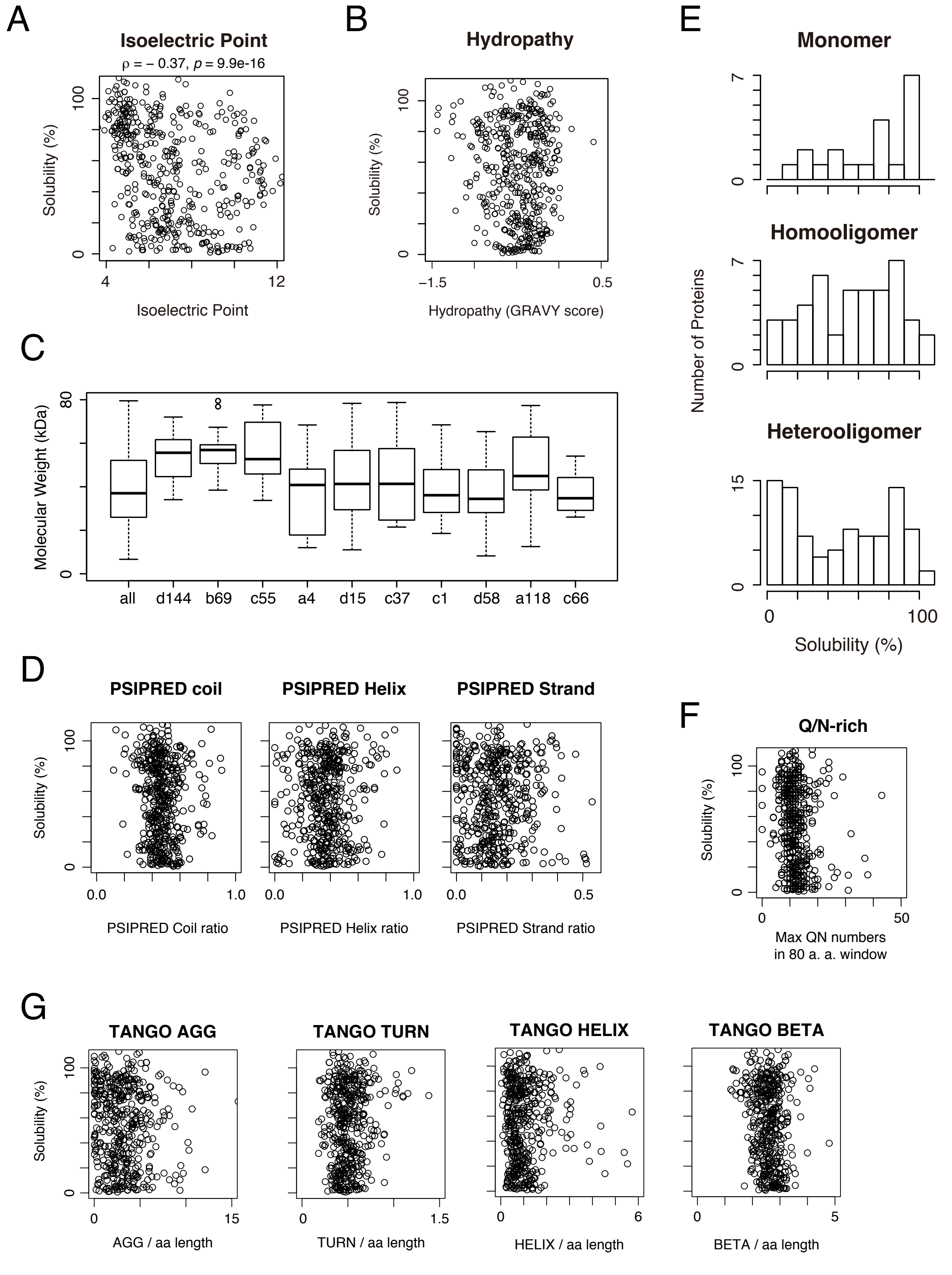

Supplementary Figure S2. Comparison of the aggregation propensity with physicochemical properties and structural parameter.

(A) A scatter plot of the solubility evaluated at 37 °C and isoelectric point. The value ρ indicates Spearman's rank correlation coefficient ($p = 9.9 \times 10^{-16}$). (B) A scatter plot of the solubility evaluated at 37 °C and the GRAVY score, the index of hydrophobicity. (C) A boxplot of the molecular weight

in each SCOP fold group. The names of each SCOP fold are as follows: d.144; Protein kinase-like (PK-like), b.69; 7-bladed beta-propeller, c.55; Ribonuclease H-like motif, a.4; DNA/RNA-binding 3-helical bundle, d.15; beta-Grasp (ubiquitin-like), c.37; P-loop containing nucleoside triphosphate hydrolases, c.1; TIM beta/alpha-barrel, d.58; Ferredoxin-like, a.118; alpha-alpha superhelix, c.66; S-adenosyl-L-methionine-dependent methyltransferases. (D) Scatter plots of the solubility evaluated at 37 °C and the ratio of secondary structure regions predicted by the PSIPRED algorithm. (E) Histograms of the solubility evaluated at 37 °C for each oligomeric state. Oligomeric states were determined from the information of the SUBUNIT annotation in the UniProt database. (F) A scatter plot of the solubility evaluated at 37 °C and the amyloidogenicity based on the Q/N content. The amyloidogenicity was determined as the max number of Gln and Asn residues in 80 amino acid window. (G) Scatter plots of the solubility evaluated at 37 °C and the predicted aggregation propensity calculated by TANGO algorithm.

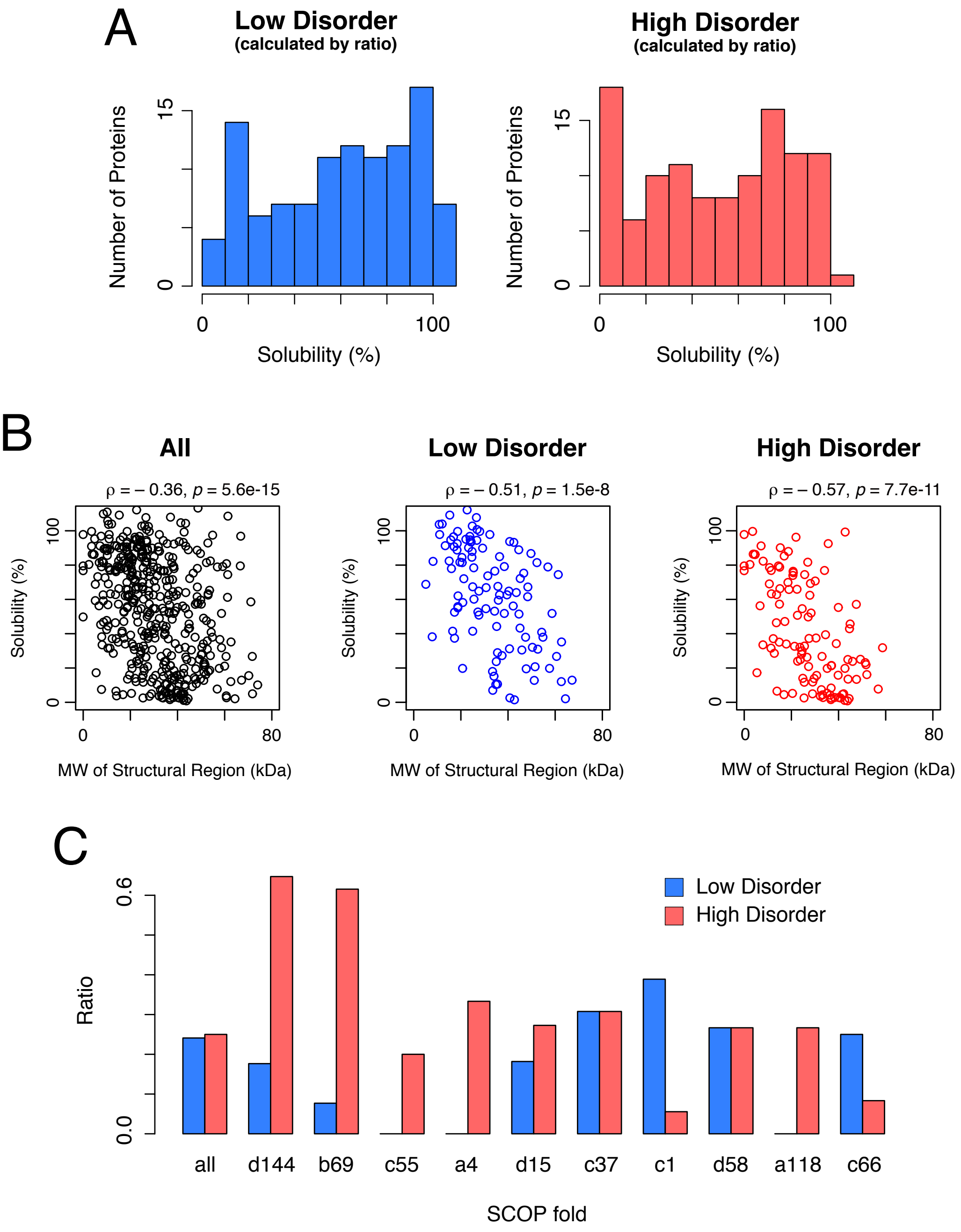

Supplementary Figure S3. Relationship between the aggregation propensity and intrinsically disordered regions.

(A) Histograms of the solubility evaluated at 37 °C for low and high disorder protein groups. The proteins in the low and high disorder groups were defined by the ratio of the disorder region to the full length that is below the 25th percentile (4.3 %) and above the 75th percentile (32.3 %), respectively. The *p* value of Wilcoxon rank-sum test between the two distributions was 0.028. (B) Scatter plots of the solubility evaluated at 37 °C and the molecular weight of the predicted structural region. Structural regions were defined as the regions that were not predicted as IDRs by DICHOT algorithm. (C) A bar chart of the ratio of the proteins in the low and high disorder groups in each SCOP fold group. The names of each SCOP fold are as follows: d.144; Protein kinase-like (PK-like), b.69; 7-bladed beta-propeller, c.55; Ribonuclease H-like motif, a.4; DNA/RNA-binding 3-helical bundle, d.15; beta-Grasp (ubiquitin-like), c.37; P-loop containing nucleoside triphosphate hydrolases, c.1; TIM beta/alpha-barrel, d.58; Ferredoxin-like, a.118; alpha-alpha superhelix, c.66; S-adenosyl-L-methionine-dependent methyltransferases.

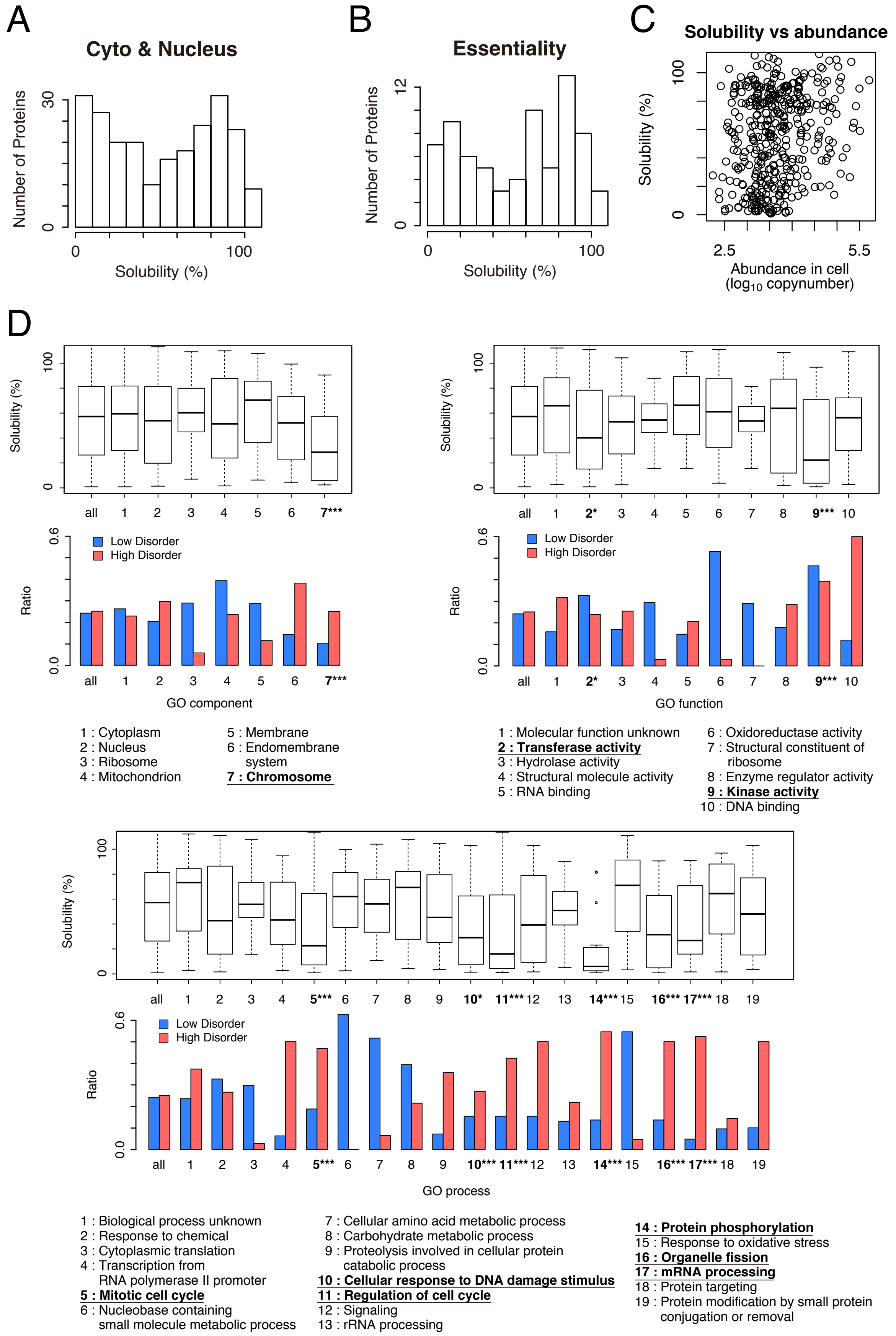

Supplementary Figure S4. Relationship between the aggregation propensity and cellular functions, essentiality, and abundance in the cell.

(A) A histogram of the solubility evaluated at 37 °C for the proteins annotated to be localized in both cytosol and nucleus. (B) A histogram of the solubility evaluated at 37 °C for the proteins annotated to be essential for cell growth. (C) A scatter plot of the solubility evaluated at 37 °C and the abundances in the cell. (D) Boxplots of the solubility evaluated at 37 °C in each Gene Ontology (GO) category. The bar charts below the boxplots indicate the ratio of the proteins in the low and high disorder groups. The names of each GO category were shown below the plots. Only the GO categories that contain more than 20 proteins were shown. *$p < 0.05$, ***$p < 0.01$, Wilcoxon rank-sum test.

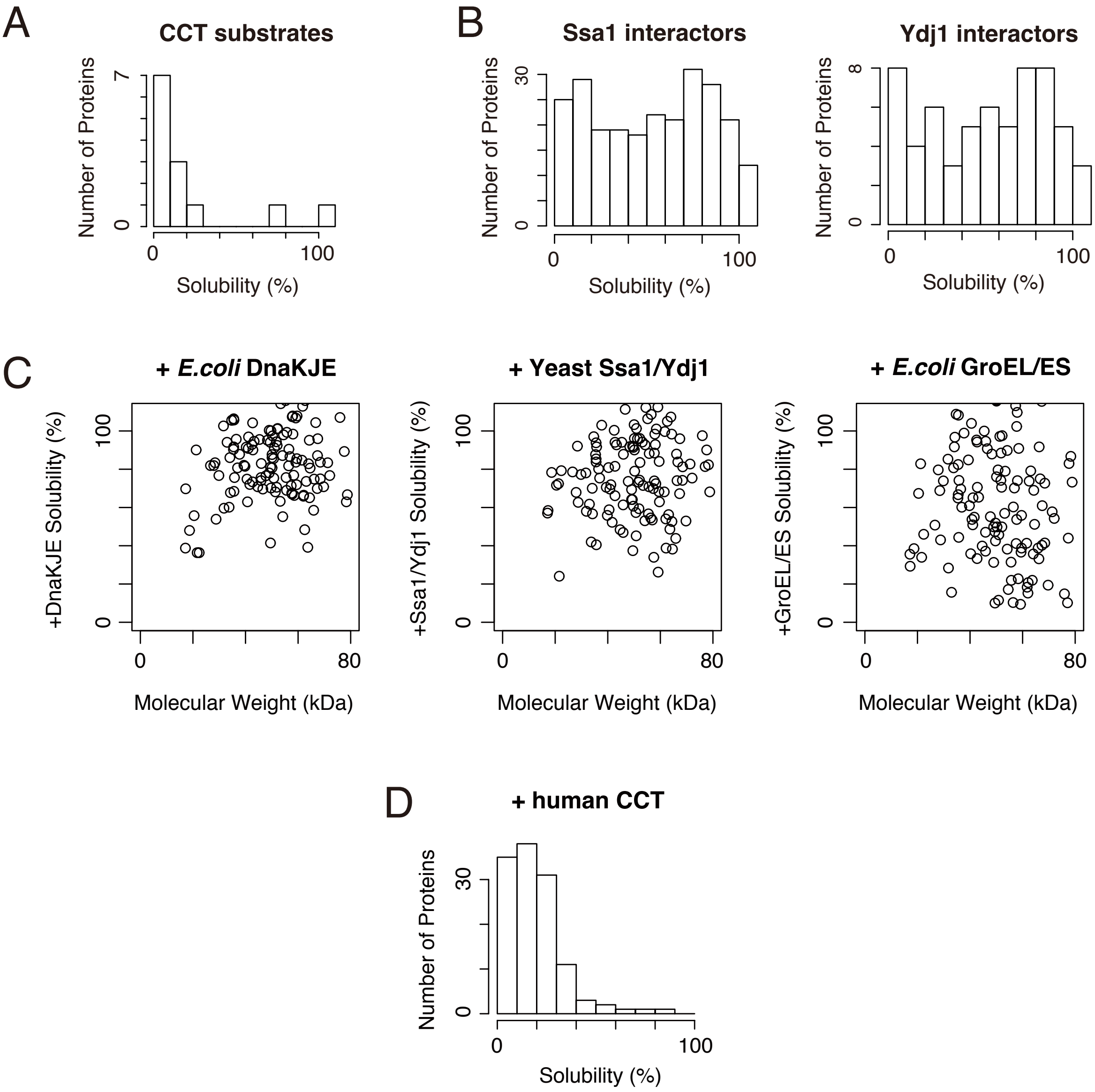

Supplementary Figure S5. The distributions of the aggregation propensity for the chaperone substrates/interactors and the comparisons between the aggregation-prevention effects of the chaperones and molecular weight.

(A) A histogram of the solubility evaluated at 37 °C for the proteins annotated as the substrates of CCT. (B) Histograms of the solubility evaluated at 37 °C for the Ssa1 or Ydj1 interacting proteins. (C) Scatter plots of the solubility in the presence of the chaperones and molecular weight. (D) A histogram of the solubility in the presence of human CCT chaperone for 124 aggregation-prone proteins, evaluated at 37 °C.

Supplementary Dataset S1. The list of 447 proteins with the evaluated solubilities and various properties. The 131 unevaluated proteins were also listed in the other sheet.

Supplementary Dataset S2. The annotation of Gene Ontology classification constructed by using GO slim mapper provided by the *Saccharomyces* Genome Database.

Supplementary Dataset S3. The list of homologous protein pairs between *E. coli* and *S. cerevisiae*.
